# Supplementary material for: Bergamot Polyphenol Extract Reduces Hepatocyte Neutral Fat by Increasing Beta-Oxidation
Source: Nutrients. 2022 Aug 20;14(16):3434. doi: 10.3390/nu14163434 (PMC9412422; doi:10.3390/nu14163434)
Supplement: Supplementary file 1 [file nutrients-14-03434-s001.zip › nutrients-1848124-supplementary.pdf]

SAMPLE REPORT

Acquisition Date/Time	06-Nov-20 11:35:27
Acquisition Method	Flavonoids cal. on Naringin
Chromera Version	4.2.0.6415
Dilution Factor	300
Report Date/Time	20-Nov-20 13:21:25
Sample Name	BPF-Gold Lot. 1802-20
Vial Number	2

BPF-Gold Lot. 1802-20 : 284:10:400:10 : 1

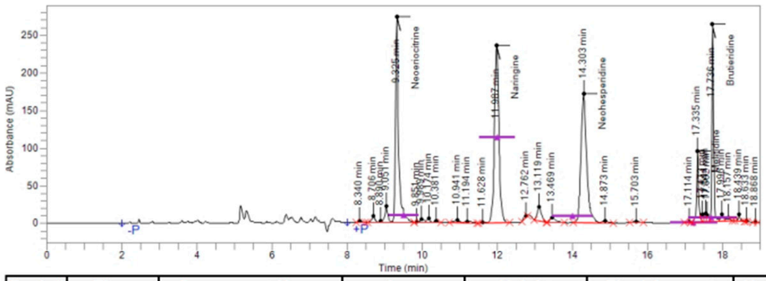

Peak #	Time	Component Name	Area	Height	Final Amount	Units
25	17.736	Brutieridine	744,325.4	263,757.9	54,533.3067	µg/g
21	17.335	Melitidine	375,035.4	94,608.6	26,946.3741	µg/g
13	11.987	Naringine	1,915,031.8	235,500.2	141,385.1452	µg/g
5	9.325	Neoeriocitrine	1,587,619.4	273,788.4	132,517.0057	µg/g
17	14.303	Neohesperidine	1,816,561.2	171,299.2	134,119.0245	µg/g
Total			6,438,573.2		489,500.8563	

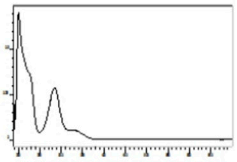

Peak: 5, 9.325min, Neoeriocitrine

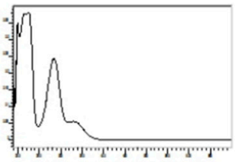

Peak: 13, 11.987min, Naringine

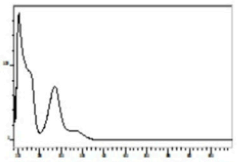

Peak: 17, 14.303min, Neohesperidine

Channel Name: 284:10:400:10

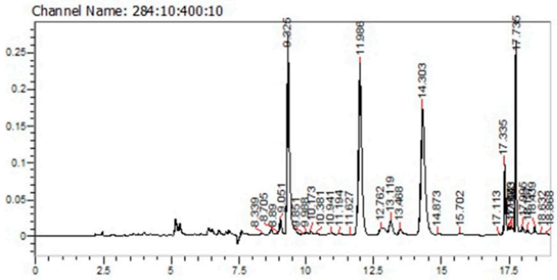

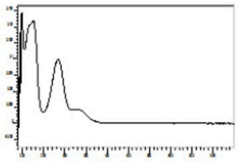

Peak: 21, 17.335min, Melitidine

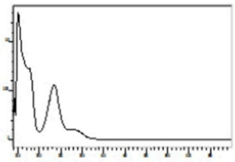

Peak: 25, 17.736min, Brutieridine

Figure S1: Bergamot PF extract analysis of by HPLC-UV

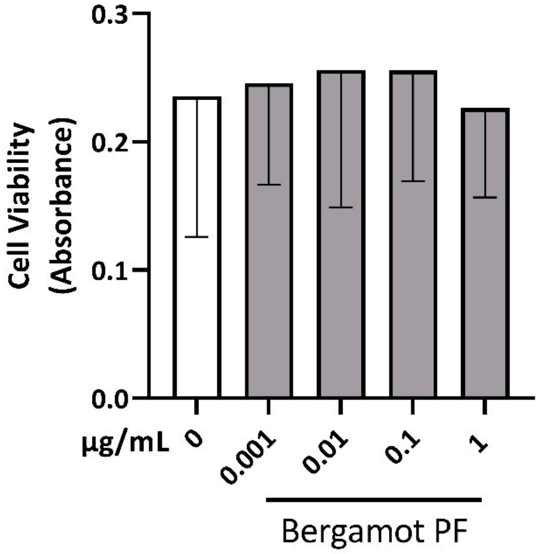

Figure S2: Bergamot PF does not increase viability of McA Rh-7777 cells. Semi-confluent cultures of rat hepatoma cell line (McA Rh-7777) incubated with bergamot PF extract (0.001- 0.01- 0.1- 1 µg/ml) for 24h. (A) Cell viability determined by MTT assay. Data are represented as mean ± SD of three independent experiments. *Abbreviations: PF, polyphenol fraction; MTT assay, 3-(4,5-dimethylthiazol-2-yl)-2,5- diphenyltetrazolium bromide assay; SD, standard deviation*

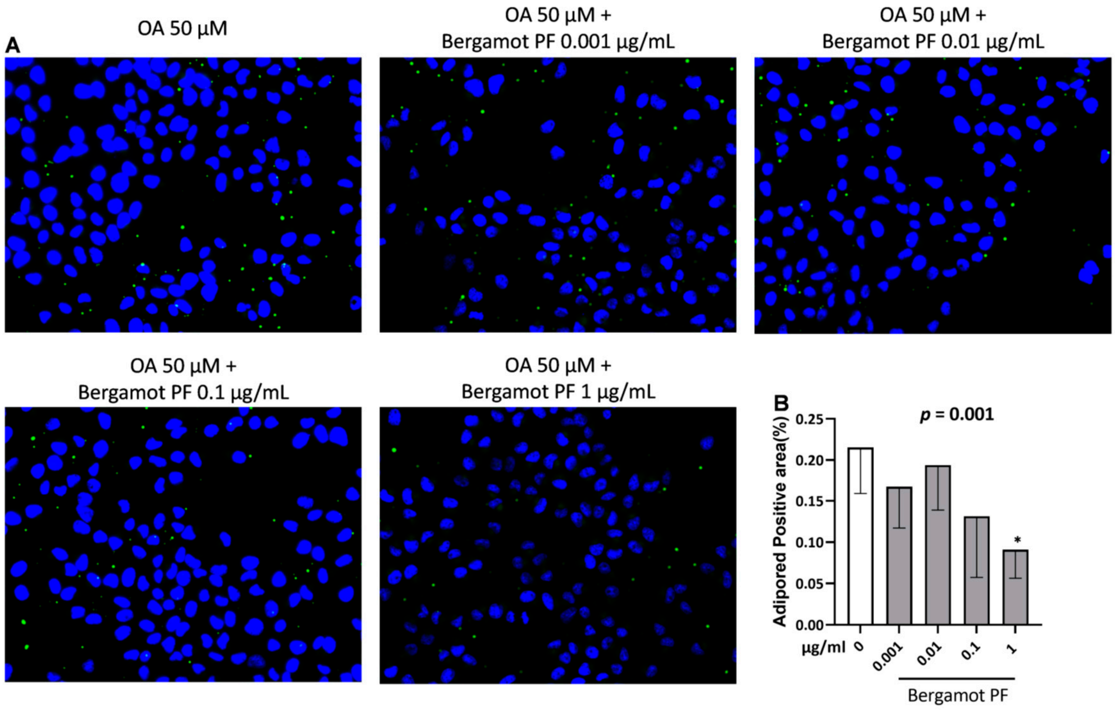

Figure S3: Incubation with bergamot PF extract **reduces triglyceride content in 2D cultured hepatocytes.** (A) Rat hepatoma cell line McA Rh7777 was cultured in 2D and incubated with 50 µM of oleic acid and different concentrations of bergamot PF extract (0.001- 0.01- 0.1 - 1 µg/ml) in regular medium without FBS for 24h. Triglyceride content was visualized by Adipored Assay kit. (B) Adipored area quantified per DAPI stained nuclei by Image J showed a dose dependent reduction with a statistically significant result also at dose 1 µg/ml. Data shown as mean ± SD of three independent experiments. Statistical analysis: Student`s t-test vs. 0 *$p<0.05$. Linear regression $p=0.001$ *Abbreviations: PF, polyphenols fraction; FBS, fetal bovine serum; OA, oleic acid; SD, standard deviation; 2D, two dimensional.*

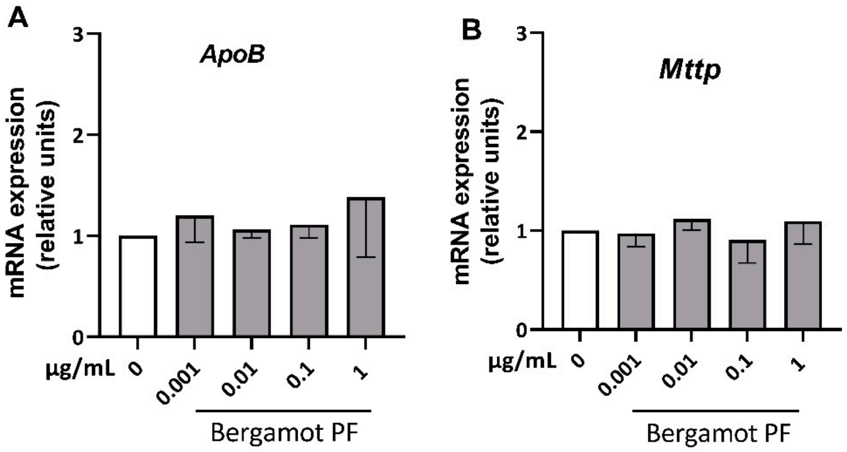

Figure S4: Bergamot PF extract does not influence the levels expression of ApoB and Mttp on McA Rh-7777 cells. Semi-confluent cultures of rat hepatoma cell line (McA Rh-7777) were incubated with 50 µM of oleic acid and different concentrations of Bergamot PF extract (0.001- 0.01- 0.1 - 1 µg/ml) in regular medium without FBS for 24h. Then, mRNA expression levels of (A) *ApoB* and (B) *Mttp* were measured by Real-time PCR. Data were analysed using the 2^-ΔΔcq method and normalized to *β-Actin*. Data are represented as mean ± SD of three independent experiments. Abbreviations: PF, polyphenol fraction; ApoB, Apolipoprotein B; Mttp, Microsomal triglyceride transfer protein; SD, standard deviation.

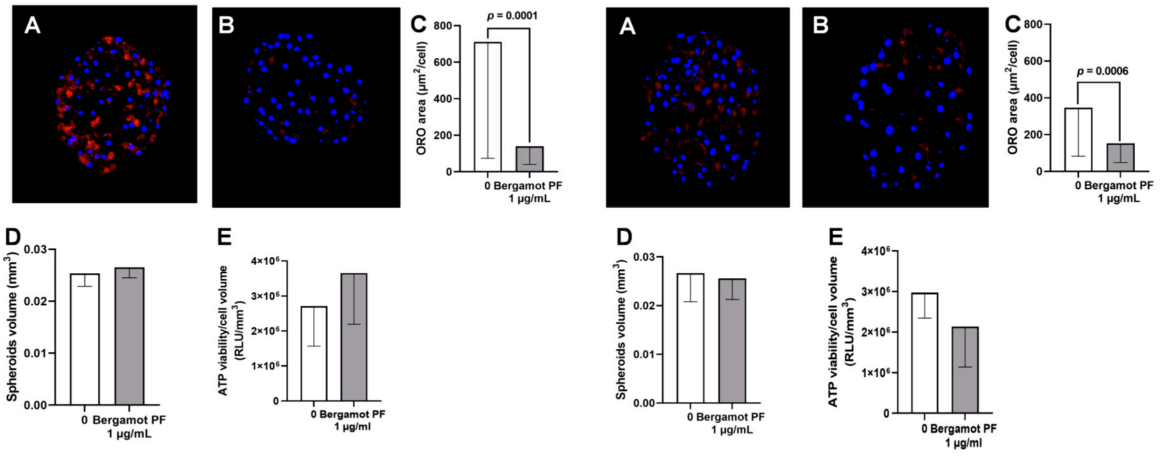

Figure S5: Treatment with 1µg/mL bergamot PF extract reduces intracellular lipid content in human liver organoid. (A) Human liver organoid was cultured as 3D spheroids for a total of 7 days. Initially, 48 hours after seeding the media replacement every 48 hours. Objective 20X. (B) Human liver organoid was cultured as 3D spheroids for a total of 7 days. Initially,48 hours after seeding the media was supplemented with 1µg/mL bergamot PF extract for an additional 5 days with media replacement every 48 hours. Objective 20X. (D)The average volume was calculated measuring their long and short diameter by ZEN 2.3 Lite software (Zeiss). (E) For both spheroids, cellular ATP levels remained stable between the experimental groups. (C) Intracellular lipid content measured by Oil red-O staining showed a significant reduces after incubation with bergamot PF extract. Data shown as mean ± SD in all groups. Statistical analysis: Mann-Whitney non-parametric test vs 0. Abbreviations: PF, polyphenols fraction; 3D, three dimensional; SD, standard deviation.

Table S1. Real time-PCR primer sequences.

Gene	Forward	Reverse
Srebp-1c	5'-CCAGCCTTTGAGGATAACCA -3'	5'-TGCAGGTCAGACACAGGAAG-3'
Acox1	5'-TCAACAGCCCAACTGTGACTTCCATTA-3'	5'-TCAGGTAGCCATTATCCATCTCTTCA-3'
Pparα	5'-ATCCACGAAGCCTACC-3'	5'-CACACCGTACTTTAGCAAG-3'
β-ctin	5'-GCCCTGAGGCACTCTTCCA-3'	5'-TTGCGGATGTCCACGTCA-3'

Table S2. Real time-PCR probes.

Gene	Probes
Ucp2	Rn01754856_m1
Atg7	Rn01492725_m1
ApoB	Rn01499054_m1
Mttp	Rn01522963_m1
β-Actin	Rn00667869_m1
SREBP-1C	Hs01088691_m1
ACOX1	Hs01074241_m1
PPARA	Hs00947539_m1
UCP2	Hs01075227_m1
ATG7	Hs00893766_m1
APOB	Hs01071209_m1
B-ACTIN	Hs01060665_g1
